# Application of serum STIM1, AOPPS, and urinary NGAL, AGT in the diagnosis of hypertensive nephropathy

**DOI:** 10.1080/0886022X.2025.2515527

**Published:** 2025-06-24

**Authors:** Baohua Li, Chunsheng Wang, Deng Ouyang, Haofeng Xu, Zhile Wu, Xin Yang

**Affiliations:** ^a^ Department of General Practice, The First Affiliated Hospital of Guangzhou Medical University, Guangzhou, Guangdong Province, China; ^b^ Department of Hemodialysis, Guangzhou Guanggang New City Hospital, Guangzhou, Guangdong Province, China; ^c^ Department of Neurology, The Second People’s Hospital of Baiyun, Guangzhou, Guangdong Province, China; ^d^ Department of Geriatrics, The First Affiliated Hospital of Guangzhou Medical University, Guangzhou, Guangdong Province, China

**Keywords:** Hypertensive nephropathy, STIM1, AOPPs, NGAL, AGT, early detection

## Abstract

**Objective:**

We aim to explore the diagnostic value of early detection of serum stromal interaction molecule 1 (STIM1), advanced oxidation protein products (AOPPs), urinary neutrophil gelatinase-associated lipocalin (NGAL), and angiotensinogen (AGT) in hypertensive nephropathy (HN).

**Methods:**

A retrospective study was conducted on 123 patients with primary hypertension. Based on the diagnostic criteria for HN, patients were divided into the HN group (*n* = 58) and simple hypertension group (*n* = 65). Additionally, 60 healthy individuals undergoing routine physical examinations were selected as the control group. The concentrations of serum STIM1, AOPPs, and urinary NGAL and AGT were assessed.

**Results:**

The HN group exhibited higher levels of serum STIM1, AOPPs, and urinary NGAL and AGT than the simple hypertension and control groups. Pearson correlation analysis revealed that serum STIM1 and AOPPs were positively correlated with BUN, UN, and SCr and negatively correlated with eGFR; urinary NGAL and AGT were positively correlated with blood urea nitrogen, uric acid, and serum creatinine and negatively correlated with eGFR (all *p* < 0.05). Logistic regression analysis displayed a correlation between HN and serum STIM1 (OR: 1.019; 95%CI: 1.049-1.148), AOPPs (OR: 1.312; 95%CI: 1.129-1.526), and AGT (OR: 1.436; 95%CI: 1.183-1.742) (*p* < 0.05). ROC curve analysis demonstrated that the combined detection of serum STIM1, AOPPs, and urinary NGAL, AGT had a higher AUC for predicting HN compared to individual tests.

**Conclusion:**

Detection of serum STIM1, AOPPs, and urinary NGAL and AGT has diagnostic value in HN, providing a reference for early diagnosis and timely treatment of HN.

## Introduction

Hypertensive nephropathy (HN) is a major complication of chronic hypertension, resulting in kidney damage marked by podocyte injury, cell death, and eventual glomerulosclerosis [1]. It is a major factor in the development of chronic kidney disease (CKD) and end-stage renal disease (ESRD), significantly impacting global morbidity, mortality, and healthcare expenses [2]. With hypertension affecting around 30% of the global population, understanding and addressing its renal consequences is critical [3]. The pathogenesis of HN involves complex interactions among hemodynamics, inflammation, and fibrosis. Long-term hypertension leads to increased intraglomerular pressure, exerting mechanical stress on glomerular capillaries and subsequently causing endothelial dys­function. The renin-angiotensin-aldosterone system (RAAS) is central to this process, promoting vasoconstriction, sodium retention, and vascular remodeling, thereby exacerbating kidney damage [4]. However, early diagnosis of HN remains a crucial topic due to its nonspecific diagnostic criteria and clinical overlap with other kidney conditions [5].

Currently used biomarkers of kidney function impairment, such as blood urea nitrogen (BUN), serum creatinine (SCr), and cystatin C [6], often only show abnormalities after kidney damage has reached a certain level and are susceptible to various factors [7–9]. They have limitations in early prediction or diagnosis of kidney injury. Thus, the search for more sensitive and specific biomarkers to improve the early diagnosis rate of HN remains a hot research topic.

Promising candidates include serum stromal interaction molecule 1 (STIM1) [10], advanced oxidation protein products (AOPPs) [11], urinary neutrophil gelatinase-associated lipocalin (NGAL) [12], and angiotensinogen (AGT) [13]. STIM1 is a transmembrane protein with its Ca2+ sensing domain facing the lumen of the endoplasmic reticulum. Ca2+ release plays a vital role in signal transduction in the kidney [14]. Recent studies have found a correlation between STIM1 mutations and HN [15]. It regulates the phenotype and function of podocytes, affecting the progression of kidney disease [10]. Additionally, AOPPs are cross-linked protein products containing dityrosine, which produce albumin and protein oxidation, structurally resemble advanced glycation end products, and also exhibit biological activity. Their levels are elevated in patients with diabetes, hypertension, and atherosclerosis [11]. Giovanni et al. suggested that measuring oxidative stress levels with AOPPs appears to be a promising new indicator of kidney function impairment [11]. On the other hand, NGAL is a protein secreted by activated neutrophils [16]. Increased urinary NGAL may reflect kidney injury [12]. Its levels are correlated with inflammatory status in hypertension [16]. Furthermore, AGT is a serine protease family protein mainly produced by the liver, systematically processed by proteases of the RAAS to produce hormone peptides [17].

In light of this, these biomarkers capture key pathophysiological processes, including oxidative stress, inflammation, and changes in renal hemodynamics, which are crucial for the progression of HN. Therefore, we combined serum STIM1/AOPP and urinary NGAL/AGT for the first time, aiming to explore the relationship between these biomarkers and HN, in order to provide new ideas and methods for the early diagnosis and effective treatment of HN. We hypothesize that compared with conventional biomarkers, the combination of serum STIM1/AOPP and urinary NGAL/AGT can improve the diagnostic accuracy of HN.

## Materials and methods

### Ethical approval

All experimental procedures were approved by the Medical Ethics Committee of The First Affiliated Hospital of Guangzhou Medical University. The patient has been notified about the information contained in the consent form.

### Basic information

General information was collected from 123 patients with primary hypertension who were diagnosed and treated in The First Affiliated Hospital of Guangzhou Medical University between January 2022 and March 2023. Based on the diagnostic criteria for HN, the patients were categorized into two groups: the HN group (*n* = 58) and the simple hypertension group (*n* = 65). Furthermore, 60 healthy individuals who underwent physical checkups at our hospital’s health examination center during the same period were chosen as the control group.

Inclusion criteria: (1) Patients diagnosed with primary hypertension according to the ‘Chinese Guidelines for the Prevention and Treatment of Hypertension (2018 Revised Edition)’, defined as having a systolic blood pressure ≥ 140 mmHg and/or diastolic blood pressure ≥ 90 mmHg; Diagnostic criteria for HN: patients with an estimated glomerular filtration rate (eGFR) < 60 mL/min/1.73 m^2^ and a urine albumin-to-creatinine ratio (ACR) ≥ 30 mg/g [18,19]; (2) patients aged between 50 and 80 years; (3) patients who have signed the informed consent form.

Exclusion criteria: (1) Patients with primary kidney disease or eGFR < 15 mL/min/1.73 m^2^; (2) patients with concomitant malignancies, diabetes, or other cardiovascular and cerebrovascular diseases; (3) patients with autoimmune diseases, etc.; (4) patients with incomplete clinical data.

A total of 180 patients were prescreened for this study, with 150 proceeding to formal screening. Based on the exclusion criteria, 27 of the 150 patients were excluded during formal screening, leaving a final sample of 123 patients.

### Data collection

General data of the study subjects were collected, including sex, age, body mass index (BMI), blood pressure (systolic and diastolic), duration of hypertension, duration of HN, history of underlying diseases (diabetes, hyperlipidemia), and renal function indicators, including BUN, uric acid (UA), SCr, and eGFR.

### Sample collection and testing

All participants were instructed to fast for 8 h, avoiding both food and water. Peripheral venous blood (5 mL) and morning urine (20 mL) were collected from primary hypertension patients on the morning of the second day after enrollment, and from healthy individuals on the day of their physical examination. The samples were centrifuged at 3000 rpm for 5 min, and the supernatant was collected. The time from sample collection to centrifugation was (18.63 ± 3.68) minutes. Serum STIM1 and AOPPs levels, as well as urinary NGAL and AGT levels, were measured using enzyme-linked immunosorbent assay (ELISA). All kits were obtained from Senbei Biotechnology Co., Ltd., Nanjing, China. The sensitivity (detection limit: 5 pg/mL) and precision (intra-assay CV = 3%/inter-assay CV = 8%) of the ELISA kits were validated. The testing procedures were strictly followed according to the manufacturer’s instructions.

### Statistical analysis

The normality of data distribution was assessed using the Shapiro-Wilk test, Kolmogorov-Smirnov test, or Q-Q plot in SPSS 22.0. For normally distributed data, results were expressed as mean ± standard deviation. Comparisons were conducted by the independent samples t-test. When comparing multiple groups, a one-way analysis of variance (ANOVA) was employed if the data satisfied the assumptions of normality and homogeneity of variances, with least significant difference used for pairwise comparisons. In cases where variances were unequal, Welch’s ANOVA or the non-parametric Kruskal-Wallis H test was utilized. For data that did not conform to a normal distribution, the median (M) and interquartile range (IQR) were used for description. Non-parametric methods, such as the Mann-Whitney U test for comparing two independent groups or the Kruskal-Wallis H test for multiple groups, were applied for intergroup comparisons.

Categorical data were presented as frequencies or percentages (%), and group comparisons were conducted using the chi-square (χ^2^) test. Variables that demonstrated significant differences in univariate analysis were incorporated as independent variables in logistic regression to identify factors associated with HN. Receiver operating characteristic (ROC) curves were constructed, and the area under the curve (AUC) was computed to assess the predictive performance of serum STIM1, AOPPs, urinary NGAL, and AGT, both individually and in combination, for HN. Pearson correlation analysis was employed to assess correlations. A P-value of less than 0.05 was deemed statistically significant.

## Results

### General information

As shown in Table 1, the simple hypertension group included 34 males and 31 females, aged 50-78 years, with a mean age of (62.29 ± 8.24) years; the HN group included 32 males and 26 females, aged 52-80 years, with a mean age of (63.28 ± 9.04) years; and the control group included 33 males and 27 females, aged 50-79 years, with a mean age of (62.98 ± 9.27) years. In the three groups, no significant differences were found regarding sex, age, or BMI (*p* > 0.05) (Table 1).

**Table 1.** Comparison of clinical data among the three groups of patients.

Item	Control group (n = 60)	Simple hypertension group (*n* = 65)	HN group (*n* = 58)	Value	*P*
Sex (male/female)	33/27	34/31	32/26	0.131	0.937
Age (years)	62.98 ± 9.27	62.29 ± 8.24	63.28 ± 9.04	0.203	0.817
Body mass index (kg/m²)	24.95 ± 2.01	24.93 ± 1.99	24.95 ± 1.98	0.002	0.998
Systolic blood pressure (mm Hg)	114.53 ± 3.53	149.18 ± 4.12^a^	149.74 ± 3.90^a^	1643.908	<0.001
Diastolic blood pressure (mm Hg)	89.08 ± 3.67	114.28 ± 3.92a	114.57 ± 3.96a	871.764	<0.001
Duration of hypertension (years)	0	12.15 ± 7.42a	11.57 ± 7.74a	73.958	<0.001
Diabetes [n (%)]	2 (3.33)	55 (84.62)a	53 (91.38)a	120.594	<0.001
Hyperlipidemia [n (%)]	0	5 (7.69)a	12 (20.69)a	15.286	<0.001
BUN (mmol/L)	4.31 ± 0.54	6.96 ± 0.42	13.58 ± 1.56ab	129.261	<0.001
UN (mmol/L)	244.83 ± 77.36	244.69 ± 78.97	471.38 ± 59.66 ab	202.179	<0.001
SCr (mg/dL)	63.56 ± 14.67	98.22 ± 14.71	293.72 ± 14.41 ab	161.557	<0.001
eGFR mL/(min × 1.73 m^2^)	101.96 ± 4.78	96.37 ± 4.79	46.81 ± 5.29ab	439.504	<0.001
STIM1 (ng/L)	308.80 ± 6.65	397.30 ± 13.21a	438.33 ± 34.14 ab	527.504	<0.001
AOPPs (umol/L)	23.15 ± 7.42	23.12 ± 7.00	30.86 ± 6.19 ab	24.837	<0.001
NGAL (pg/mL)	58.60 ± 4.08	120.20 ± 19.87a	147.91 ± 34.72 ab	235.396	<0.001
AGT (μg/g)	25.92 ± 5.02	26.14 ± 4.71	32.47 ± 4.94 ab	34.347	<0.001

Note: HN, hypertensive nephropathy; BUN, blood urea nitrogen; UA, uric acid; SCr, serum creatinine; eGFR, estimated glomerular filtration rate; STIM1, serum stromal interaction molecule 1; AOPPs, advanced oxidation protein products; NGAL, urinary neutrophil gelatinase-associated lipocalin; AGT, angiotensinogen. Normally distributed measurement data are expressed as mean ± standard deviation. Compared with the control group, *^a^P* < 0.05; compared with the simple hypertension group; *^b^P* < 0.05.

### Serum and urinary biomarker levels

The HN group showed significantly higher levels of serum STIM1 and AOPPs, as well as urinary NGAL and AGT, compared to both the simple hypertension group and the control group (*p* < 0.05). Additionally, the simple hypertension group had elevated levels of STIM1 and NGAL compared to the control group (*p* < 0.05) (Table 1).

### Pearson correlation analysis

Pearson correlation analysis was conducted to examine the relationships between serum STIM1, AOPPs, urinary NGAL, AGT, and renal function indicators. The findings revealed that serum STIM1 and AOPPs were positively correlated with BUN, UN, and SCr (*r* = 0.626/0.538, 0.569/0.502, 0.614/0.049, respectively; *p* < 0.05) and negatively correlated with eGFR (r=-0.604/-0.495, *p* < 0.05); urinary NGAL and AGT were positively correlated with BUN, UN, and SCr (*r* = 0.456/0.509, 0.441/0.493, 0.438/0.542, respectively; *p* < 0.05) and negatively correlated with eGFR (r = −0.432/-0.547, *p* < 0.05) (Table 2).

**Table 2.** Correlation analysis of serum STIM1, AOPPs, and urinary NGAL, AGT, and renal function indicators in the HN group.

Indicator	Statistical value	STIM1	AOPPs	NGAL	AGT
BUN	r	0.626	0.538	0.456	0.509
	*P*	<0.001	<0.001	<0.001	<0.001
UA	r	0.569	0.502	0.441	0.493
	*P*	<0.001	<0.001	<0.001	<0.001
SCr	r	0.614	0.49	0.438	0.542
	*P*	<0.001	<0.001	<0.001	<0.001
eGFR	r	−0.604	−0.495	−0.432	−0.547
	*P*	<0.001	<0.001	<0.001	<0.001

Note: HN, hypertensive nephropathy; BUN, blood urea nitrogen; UA, uric acid; SCr, serum creatinine; eGFR, estimated glomerular filtration rate; STIM1, serum stromal interaction molecule 1; AOPPs, advanced oxidation protein products; NGAL, urinary neutrophil gelatinase-associated lipocalin; AGT, angiotensinogen.

### Logistic regression analysis

Using the occurrence of HN as the dependent variable, we conducted the logistic regression analysis with serum STIM1, AOPPs, urinary NGAL, and AGT as independent variables. The findings indicated a correlation between HN and serum STIM1 (OR: 1.019; 95%CI: 1.049-1.148), AOPPs (OR: 1.312; 95%CI: 1.129-1.526), and AGT (OR: 1.436; 95%CI: 1.183-1.742) (*p* < 0.05) (Table 3).

**Table 3.** Logistic regression analysis of risk factors for HN in patients.

Variable	β value	SE value	Wald value	*P*	OR(95%CI)
STIM1	0.093	0.023	16.241	<0.001	1.019 (1.049-1.148)
AOPPs	0.272	0.770	12.459	<0.001	1.312 (1.129-1.526)
NGAL	−0.008	0.018	0.183	0.669	0.992 (0.958-1.028)
AGT	0.362	0.099	13.441	<0.001	1.436 (1.183-1.742)

Note: HN, hypertensive nephropathy; STIM1, serum stromal interaction molecule 1; AOPPs, advanced oxidation protein products; NGAL, urinary neutrophil gelatinase-associated lipocalin; AGT, angiotensinogen.

### ROC curve analysis

**Figure 1.**
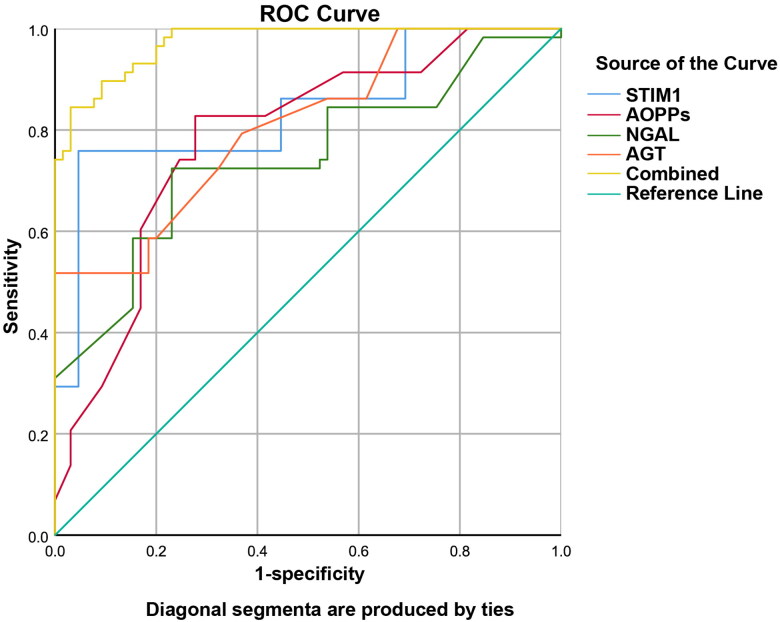
ROC Curves of STIM1, AOPPs, NGAL, AGT, and their combination in predicting the occurrence of HN.

The ROC curve was drawn, and the AUC was calculated. The results showed that the AUC for the combined detection was 0.973 (sensitivity: 84.50%, specificity: 96.90%, 95%CI: 0.951-0.994), which was higher than that for serum STIM1 alone (AUC = 0.837, sensitivity: 75.90%, specificity: 95.40%, 95%CI: 0.762-0.911), AOPPs alone (AUC = 0.784, sensitivity: 82.80%, specificity: 72.30%, 95%CI: 0.702-0.866), urinary NGAL alone (AUC = 0.744, sensitivity: 72.40%, specificity: 76.90%, 95%CI: 0.655-0.833), and AGT alone (AUC = 0.807, sensitivity: 51.70%, specificity: 100%, 95%CI: 0.732-0.882) in predicting HN (Table 4, Figure 1).

**Table 4.** Comparison of the predictive value of serum STIM1, AOPPs, and urinary NGAL, AGT for HN.

Variable	AUC	Cut-off value	Sensitivity (%)	Specificity (%)	Youden index	95%CI	*P*
STIM1	0.837	402.08	75.90	95.40	0.713	0.762-0.911	<0.001
AOPPs	0.784	26.50	82.80	72.30	0.551	0.702-0.866	<0.001
NGAL	0.744	138.50	72.40	76.90	0.493	0.655-0.833	<0.001
AGT	0.807	34.50	51.70	100.00	0.517	0.732-0.882	<0.001
Combination	0.973	/	84.50	96.90	0.814	0.951-0.994	<0.001

Note: HN, hypertensive nephropathy; STIM1, serum stromal interaction molecule 1; AOPPs, advanced oxidation protein products; NGAL, urinary neutrophil gelatinase-associated lipocalin; AGT, angiotensinogen.

## Discussion

HN is one of the most common complications of hypertension and a leading cause of end-stage renal disease in many countries [20]. Without early detection and effective management, it can result in progressive renal dysfunction and chronic kidney disease [3]. Early diagnosis and timely intervention are crucial for slowing disease progression and improving patient outcomes. This study measures the diagnostic value of combining specific biomarkers to enhance the early detection of HN. Our results indicate that the combined detection of serum STIM1, AOPPs, and urinary NGAL, AGT has application value in the diagnosis of HN, providing a reference for early diagnosis and timely treatment of HN.

Specifically, serum STIM1, AOPPs, and urinary NGAL, AGT were significantly elevated in the HN group, suggesting that these indicators can serve as potential biomarkers for HN diagnosis. In addition, there was a correlation between serum STIM1, AOPPs, urinary NGAL, AGT and renal function indicators, further validating the value of these indicators in reflecting kidney function impairment. Importantly, the study further confirmed that serum STIM1, AOPPs, and urinary AGT were correlated with HN, and the combined detection AUC was higher than that of individual indicators for predicting HN, indicating that combined detection has higher sensitivity and specificity in HN diagnosis.

Research has shown increased levels of urinary AGT and NGAL in patients with early-stage renal damage, reinforcing their utility as sensitive indicators of kidney injury [21]. It has also been reported that AOPP levels are elevated in hypertensive patients [11]; increased concentrations are associated with decreased renal function [22]. Furthermore, STIM1 levels are elevated in diabetic kidney disease [10], affecting the progression of kidney disease [10]. Our results further support these views. Notably, Fan Zhang et al.’s study showed that STIM1 expression in the kidney tissue of lupus nephritis mice is positively correlated with fibronectin, urine protein, blood BUN, and SCr levels [23]. Silvano et al. reported that AOPP is an independent predictor of renal fibrosis and rapid eGFR decline in chronic kidney disease patients [24]. Xiaoling Zhu et al. indicated that AGT gene polymorphisms are correlated with high BUN [25]. Interestingly, Søren et al.’s data showed that eGFR determines the relationship between NGAL and inflammation or renal function; in patients with normal eGFR, plasma NGAL reflects inflammation, but when eGFR decreases, plasma NGAL reflects renal function [26]. Combining these viewpoints, our study results are similar to previous findings but further support the application value of serum STIM1, AOPPs, and urinary NGAL, AGT in HN diagnosis. These biomarkers are not only closely related to the occurrence and development of HN but also improve the diagnostic accuracy of HN when used in combination.

From a mechanistic perspective, STIM1, as a key molecule in intracellular calcium signaling regulation, its elevated levels may reflect imbalances in calcium homeostasis within kidney cells, leading to impaired kidney function. Its positive correlation with BUN, UN, and SCr, as well as its negative correlation with eGFR, suggest that STIM1 may be involved in the pathological process of HN. Additionally, AOPPs are products of oxidative stress, and their elevated levels reflect an enhanced oxidative stress state in the body. The correlation between AOPPs and HN in this study suggests that oxidative stress may play a significant role in HN. Meanwhile, NGAL and AGT are known biomarkers of kidney injury, and their increased levels in urine reflect structural damage and functional decline of the kidney. The correlation between urinary NGAL, AGT, and renal function indicators in this study, along with their significant elevation in the HN group, further confirms their value in HN diagnosis. In terms of advantages, combined detection can comprehensively consider changes in multiple biomarkers, improving diagnostic accuracy. Different biomarkers may reflect different aspects of HN, and combined detection can more comprehensively assess the degree of kidney damage and functional status. Importantly, this study is the first to combine the four biomarkers of serum STIM1, AOPPs, and urinary NGAL, AGT for HN diagnosis, which is novel. The significant improvement in diagnostic accuracy through combined detection provides new ideas and references for the early diagnosis of HN.

In summary, this study reveals that the combined detection of serum STIM1, AOPPs, and urinary NGAL, AGT has application value in the diagnosis of hypertensive nephropathy, providing a reference for early diagnosis and timely treatment of hypertensive nephropathy. However, this study has some limitations. For example, sample size calculation was not performed, and the sample size was small, with a single-center design, which may affect generalizability and introduce bias. Furthermore, this study did not adjust for confounding factors such as age, sex, and BMI. Regrettably, this study lacks external validation, and the stability of its findings needs further confirmation. In clinical use, these biomarkers can serve as auxiliary diagnostic tools when eGFR/ACR results are uncertain or controversial. However, eGFR/ACR remains the gold standard for assessing renal function, with a broad application basis and clinical value. Although these biomarkers have high diagnostic accuracy, they still need further validation and optimization to ensure their reliability and stability.

In future research, the sample size should be expanded, and a multicenter design should be adopted to improve the generality and applicability of the study results. Additionally, potential confounding factors should be adjusted for during data analysis to enhance the accuracy of the study results; the study results should be validated in different populations or independent samples to assess their reliability and stability. Importantly, further studying the mechanism of action of serum STIM1, AOPPs, and urinary NGAL, AGT in HN is also necessary, providing a theoretical basis for clinical diagnosis and treatment. In clinical practice, when physicians use these biomarkers for diagnosis, they should consider the patient’s medical history, clinical manifestations, and other laboratory test results for a comprehensive assessment. Regular monitoring of changes in the levels of these biomarkers and formulating individualized treatment plans based on the patient’s specific condition and biomarker levels can improve treatment effectiveness and the patient’s quality of life.
